# Population-specific signatures of intra-individual mitochondrial DNA heteroplasmy and their potential evolutionary advantages

**DOI:** 10.1038/s41598-019-56918-6

**Published:** 2020-01-14

**Authors:** Yaron Tikochinski, Carlos Carreras, Gili Tikochinski, Sibelle T. Vilaça

**Affiliations:** 1 Faculty of Marine Sciences, Ruppin Academic Center, Michmoret, Israel; 2 Department of Genetics, Microbiology and Statistics and IRBio, University of Barcelona, Diagonal 643, 08028 Barcelona, Spain; 3 65a Hanasi, Herzliya, Israel; 4 Leibniz Institute for Zoo and Wildlife Research, Department of Evolutionary Genetics, Berlin, Germany; 5 Present Address: Department of Life Sciences and Biotechnology, University of Ferrara, Ferrara, Italy

**Keywords:** Evolutionary genetics, Conservation genomics, Population genetics

## Abstract

Heteroplasmy is the existence of more than one mitochondrial DNA (mtDNA) variant within a cell. The evolutionary mechanisms of heteroplasmy are not fully understood, despite being a very common phenomenon. Here we combined heteroplasmy measurements using high throughput sequencing on green turtles (*Chelonia mydas*) with simulations to understand how heteroplasmy modulates population diversity across generations and under different demographic scenarios. We found heteroplasmy to be widespread in all individuals analysed, with consistent signal in individuals across time and tissue. Significant shifts in haplotype composition were found from mother to offspring, signalling the effect of the cellular bottleneck during oogenesis as included in the model. Our model of mtDNA inheritance indicated that heteroplasmy favoured the increase of population diversity through time and buffered against population bottlenecks, thus indicating the importance of this phenomenon in species with reduced population sizes and frequent population bottlenecks like marine turtles. Individuals with recent haplotypes showed higher levels of heteroplasmy than the individuals with ancient haplotypes, suggesting a potential advantage of maintaining established copies when new mutations arise. We recommend using heteroplasmy through high throughput sequencing in marine turtles, as well as other wildlife populations, for diversity assessment, population genetics, and mixed stock analysis.

## Introduction

The mitochondrial DNA (mtDNA) is a fundamental part of the genome of eukaryotic cells as a centerpiece of its metabolic processes. Vertebrate mitochondrial DNA (mtDNA) accounts for less than 0.0001% of the total genomic content within a cell^1^ but due to its contribution to the oxidative phosphorylation, mutations can be associated with inherited diseases, cancer, and aging in humans^2,3^. The mtDNA is a small double-stranded circular genome averaging about 16 kb contained inside an organelle (the mitochondria) in multiple copies. It is estimated that each somatic cell contains at least 1000 mitochondrial genomes^4^.

In vertebrates, unlike the bi-parental inheritance in nuclear DNA, mitochondrial genomes are predominantly inherited though the maternal lineage (but see^5^). Typically, an offspring will inherit several copies of the mtDNA from its mother. If these copies slightly differ from one another, this variation is termed mitochondrial heteroplasmy and will be present in all cells of the new individual. Furthermore, due to a lack of nuclear repair enzymes in the mitochondria, the mtDNA has a high rate of spontaneous mutations and thus heteroplasmy is an inevitable phenomenon, as some of the mtDNA copies of each cell may mutate while other copies remain unchanged. It is estimated that the mtDNA substitution rate can be 20 to 100 times higher than their nuclear counterparts^6^. Therefore, both at the cellular as well as the whole organism level, a state of homoplasmy (where all mtDNA copies are identical) is rarely found and heteroplasmy containing at least two or more variants is common^7^. In humans, heteroplasmy is common in healthy individuals^8^ and can increase with age, as mutations during ones’ lifetime accumulate^9^.

Even though heteroplasmy is a common feature of mtDNA, the detection of low-frequent variants of an individual with heteroplasmy is still a challenge. However, the advent of high throughput sequencing (HTS) has facilitated the detection of heteroplasmy, given the possibility of sequencing the same fragment thousands of times, allowing the detection of these low-frequent haplotypes in heteroplasmy^8^. Using HTS and double droplet PCR, Li *et al*.^10^ and Rebolledo-Jaramillo *et al*.^8^ analyzed the level of heteroplasmy that is inherited by the offspring and used simulations to estimate the effects of the cellular bottleneck (i.e., the reduction in the number of segregating units of mitochondrial genomes during oogenesis^11^). Rebolledo-Jaramillo *et al*.^8^ showed that heteroplasmy is ubiquitous in humans, and an individual has on average one more heteroplasmic variant present at a frequency higher than 1%.

Previous studies have observed the occurrence of a severe inter-generation shift of frequency in mtDNA alleles (termed haplotypes) present in heteroplasmy^12^. This shift was assumed to be caused by the cellular bottleneck impact on the haplotype frequencies of mtDNA transmitted from mother to offspring. During oogenesis, the number of mitochondria molecules decreases drastically, thus producing a cellular bottleneck^11,13^. This mitochondrial bottleneck also prevents the accumulation of deleterious mutations that could otherwise happen given the clonal inheritance of mtDNA (i.e., Muller’s ratchet effect^14^), as new mutations would be rare by definition, and thus removed from the mtDNA genetic load by the random nature of a bottleneck^4^. Estimates of this cellular bottleneck have varied between a decrease of 200 to >1000-fold in mice^11,13,15^, and 1 to 200-fold in humans^16,17^.

A growing number of publications have demonstrated the importance of evolutionary processes within and among cytoplasms that affect the phenotypic expression of mtDNA^18^. Considering the mtDNA within a cell’s cytoplasm behaves as a population, heteroplasmy is an obligatory state between homoplasy and the generation of new haplotypes. As any population, the mtDNA molecules within a cell are subject to evolutionary processes such as selection and drift. The dynamics of heteroplasmy under neutrality depends on the mutation rate per gene per generation, and the effective number of mitochondria per cell which, given a strictly maternal inheritance, corresponds to the effective number of females in a population of individuals^19^. Consequently, in populations of highly heteroplasmic individuals, under specific conditions, the effective population size (N_e_) of the mtDNA (N_e-mt_) can surpass the N_e_ of nuclear genes (N_e-nu_) and thus the assumption of classical models without considering heteroplasmy may lead to underestimates of population parameters, such as migration rates (see^20^ for a description and examples). For example, Rebolledo-Jaramillo *et al*.^8^ concluded that the mitochondrial population after the germline bottleneck has an effective size of 30–35 mitochondria per individual. Li *et al*.^10^ estimated that an average of 9 mitogenomes are transmitted to the offspring, and that novel heteroplasmy (i.e., haplotypes not yet present in a population of individuals) is subject to negative selection. In *Drosophila*, Haag-Liautard *et al*.^21^ estimated a total effective number of mitochondrial genomes transmitted per female per generation between 13 and 42. All the aforementioned studies found significantly more heteroplasmies in the D-loop region than in protein-coding regions. Consequently, understanding the level of heteroplasmy in natural populations can lead to better estimates of effective population sizes, the effect of drift, the amount of migration among populations, and the mutation rates in the mtDNA.

Sea turtles are long living animals, and all seven extant species are of conservation concern. They have remarkable biological features that impact on the structuring and connectivity of their populations, like natal homing or long-distance migrations^22^ favoring long range colonization. Thus, the founding of new populations of marine turtles have been proposed to be driven by colonizing events of few individuals and thus potentially under strong population bottlenecks due to founder effects^23,24^. The studied mitochondrial DNA fragments exhibit low genetic diversity, possibly due to a combination of low metabolic rate and long generation time^25^, and low mutation rate^26^. The majority of population genetic studies in sea turtles used Single Nucleotide Polimorphisms (SNPs) in a fragment of the D-loop that is variable enough to define some populations, while others are largely dominated by one frequent haplotype. For instance, the Mediterranean populations of green sea turtle (*Chelonia mydas*) have one frequent D-loop haplotype (CM-A13), and a few low frequency haplotypes (one SNP variation from the common haplotype)^27^. To improve resolution in populations where the D-loop is largely monomorphic, a repetitive region of the D-loop has been suggested as an alternative to the 800 bp D-loop fragment. Using the D-loop repetitive region (mtSTR hereafter), Tikochinski *et al*.^28^ described 33 haplotypes for Israeli greens and 8% of the analyzed samples showed two or more variants (i.e., heteroplasmy) in Sanger di-deoxy sequencing chromatograms. Shamblin *et al*.^29^ used the same repetitive region to study green turtles from Brazil and detected population structure between two islands once thought to be one population. Although the use of the mtSTR region has greatly improved the resolution of sea turtles breeding stocks, all studies so far have used Sanger sequencing to define haplotypes and heteroplasmy has been reported as anecdotal without further consideration to the posterior analyses^28–30^. Sanger sequencing only allows the confidently distinct use of a maximum of two haplotypes within one individual with no esteem of its relative frequencies, consequently underestimating the number of mtSTR haplotypes within an individual. In order to assess the number of haplotypes and its frequency within a sample, studies using HTS in sea turtle’s mtSTR are needed.

In this study we performed heteroplasmy genotyping with HTS of a mtDNA segment on individuals of wild and captive green turtles with parentage relationships. Our main objective was to assess the extent of SNP as well as STR heteroplasmy in the green turtle mitochondrial genome and its temporal variation within individuals and across family lines. In order to better understand the phenomenon of heteroplasmy in natural populations, we have developed a simulation model for mtDNA inheritance that follows multi-generations mtDNA transmission, enabling us to recognize a mechanism for heteroplasmy changes within populations. Based on our findings, we propose potential evolutionary advantages of heteroplasmy.

## Results

We obtained a total of 6,391,834 paired reads among our 82 samples (average = 78,911 ± 48,706 paired reads, Supplementary Table S1), from which 5,156,178 were assigned to a haplotype after quality control (mean of 62,880 reads with a defined haplotype per sample, Table 1, Supplementary Table S1). Blanks had a negligible number of reads (324 paired reads). The synthetic oligo with the 6-7-6-4 repeat was sequenced in two different Illumina runs yielding a total of 201,000 reads, with 97.2% of all reads from both runs showing the original synthetic oligo repeat sequence. We also observed an extremely low F_ST_ value of 0.00009 between the two runs, demonstrating the robustness of the method. All samples’ reads were separated into two regions: the 127 bp flanking segment and the mtSTR segment. Identical reads (haplotypes) were grouped and haplotypes that accounted for less than 1% of the total number of reads were omitted. For all samples, the region flanking the mtSTR had the same major haplotype and no minor haplotypes above the 1% threshold. On the other hand, the mtSTR region proved to be polymorphic and heteroplasmic. We obtained a total of 36 different mtSTR haplotypes in our dataset within the Mediterranean samples. From the 36 haplotypes found in the samples from Israel, 11 were previously reported in this nesting population using Sanger sequencing, 2 were found only in foraging grounds (orphan haplotypes), 7 were reported in other Mediterranean nesting areas and 16 had not previously been found in any Mediterranean nesting area. These 11 haplotypes, previously found in Israel, correspond to a mean of 91.24% of all reads per sample, confirming a dominant population signature within the individuals composing the population. Heteroplasmy was detected in all the samples with a mean number of haplotypes per sample of 15.46 (ranging from 6 to 22) and a mean individual haplotypic diversity (Hi) of 0.3485 (ranging from 0.18 to 0.58) (Supplementary Table S1). We also sequenced a single green sea turtle from Brazil, representing a different population. This individual had the 7_12_4_4 haplotype which is the most frequent in the Brazilian population^29^ and 7 out of the 8 most frequent minor haplotypes present in mtDNA of this turtle were major haplotypes in other Brazilian turtles^29^. When combining data between the Brazilian and the Mediterranean samples, we obtained a total of 53 different mtSTR haplotypes^29^.

**Table 1:** Sampling information and general diversity indices.

Location	Name	Code	n (individuals)	Reads/sample	Hi	h
Ashkelon	Family 1	F1	25(12)	69,243	0.4033	18.08(24)
Giv’at Olga	Family 2	F2	23(12)	84,422	0.3005	16.65(25)
Hof Gador	Family 3	F3	2(1)	109,947	0.4021	13.00(17)
Hof Gador	Nest 1	N1	9(9)	47,789	0.3697	11.89(17)
Hof Gador	Nest 2	N2	11(11)	50,310	0.3618	13.27(17)
Hof Gador	Nest 3	N3	9(9)	23,730	0.2295	11.22(15)
Hof Gador	Nest 4	N4	2(2)	24,888	0.3544	18(20)
Brazil	Brazil	BR	1(1)	16,644	0.7049	17(17)
TOTAL			82(57)	62,880	0.3485	15.46(54)

Family clusters sampled for this study including their location, the code used, the number of samples (within brackets the number of individuals), the mean number of reads with assigned haplotype obtained per sample, the mean individual haplotypic diversity (Hi), and the mean number of haplotypes (h) found per sample (within brackets the total number of haplotypes).

**Figure 1:**
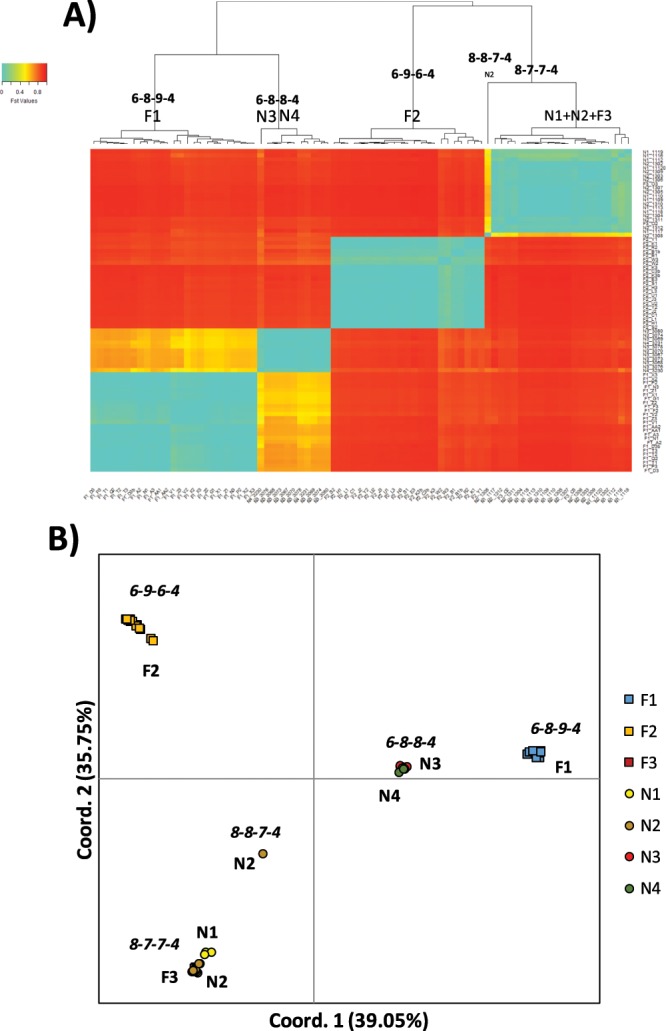
Genetic structuring among the 81 samples of known family groups including the captive animals (F1 to F3) and wild nests (N1 to N4) as detailed in Table 1. (**A**) Heatmap with dendrogram based on inter-sample pairwise F_ST_ distances. For each of the main clusters we identified the family groups included and the major haplotype among them. (**B**) Principal coordinate analysis (PCoA) of the same individuals explaining an accumulated 74.80% of the genetic variability found. For each of the main clusters we identified the family groups included and the major haplotype among them.

Significant genetic structuring (F_ST_) was found within our Mediterranean samples. Only 9 out of 3,321 p-values were non-significant after FDR correction, and always involved different samples of the same individual or samples of the same nest. The heatmap and dendrogram analysis identified five main clusters of samples (Fig. 1A), and the same result was obtained with the PCoA, explaining up to 74.8% of the genetic variability found (Fig. 1B). All the samples of a given nest or family group were included in the same cluster except one sample of the nest N2 that was assigned to a different cluster as the sole sample. Each one of the five clusters were characterized by having a different major haplotype (Fig. 1). The sample from Brazil clustered with the other Brazilian populations on the first axis of the PCoA, which explained a 63.81% of the genetic variability found, and clearly separated from Mediterranean and Caribbean populations (Fig. S1).

**Figure 2:**
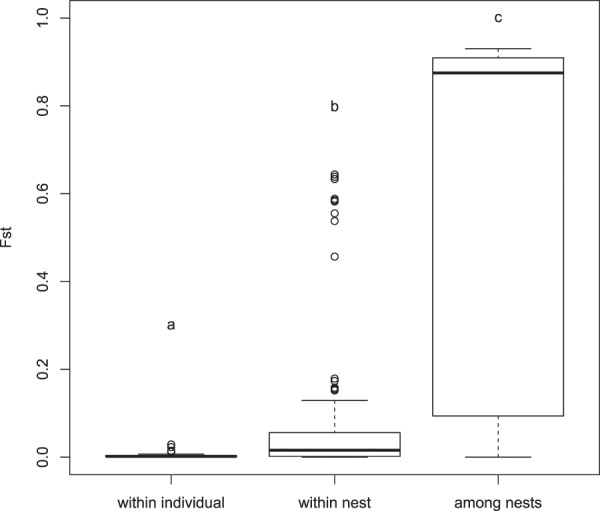
Distribution of pairwise FST genetic distances among samples considering three different levels of relationship including (i) different samples of the same individual, (ii) different samples from the same nest, and (iii) different samples from different nests. Similar letters above the bar plots represent statistically non-significantly different mean values as per the post-hoc Wilcoxon signed rank test.

The pairwise degree of differentiation was variable among samples. Samples taken from the same individual at different time periods were the least differentiated. The degree of differentiation was significantly lower than those obtained by comparing samples from different individuals of the same nest, despite having inherited their mtDNA molecules from the same individual mother (Fig. 2). The degree of differentiation between samples from different nests was highly variable and included the highest recorded values (Fig. 2).

**Figure 3:**
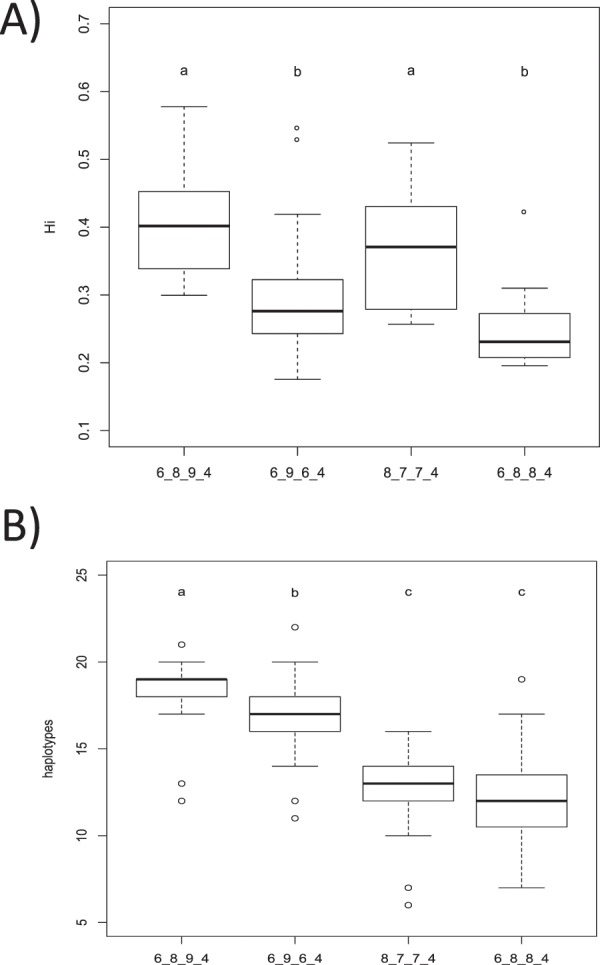
Diversity values of the 81 samples of known family groups including the captive animals (F1 to F3) and wild nests (N1 to N4) as detailed in Table 1. Samples were grouped according to the clusters detected in Fig. 1 and thus by their major haplotype. Cluster 8_8_7_4 was not represented as included only one sample, thus precluding any comparison. Diversity measures of the samples include (**A**) individual haplotype diversity (Hi), and (**B**) number of haplotypes (h). Similar letters above the bar plots represent statistically non-significantly different mean values as per the post-hoc Wilcoxon signed rank test.

The degree of heteroplasmy, measured as individual haplotype diversity or number of haplotypes, was significantly different among the families and nests sampled (Fig. S2). However, the variation within samples of the same individual was very low when compared to the variation within samples from the same nest or among samples of different nests (Fig. S3). We checked for variations in genetic variability within clusters as defined by the heatmap with the dendrogram and the PCoA (Fig. 1). The clusters with the major haplotype 6_8_9_4 and 8_7_7_4 had an individual haplotypic diversity significantly higher than the cluster with the major haplotype being 6_9_6_4 or 6_8_8_4 (Fig. 3A). Furthermore, the cluster 6_8_9_4 had a significantly higher mean number of haplotypes than the cluster with the major haplotype 6_9_6_4. The remaining two clusters had a significantly lower mean number of haplotypes per sample (Fig. 3B).

**Figure 4:**
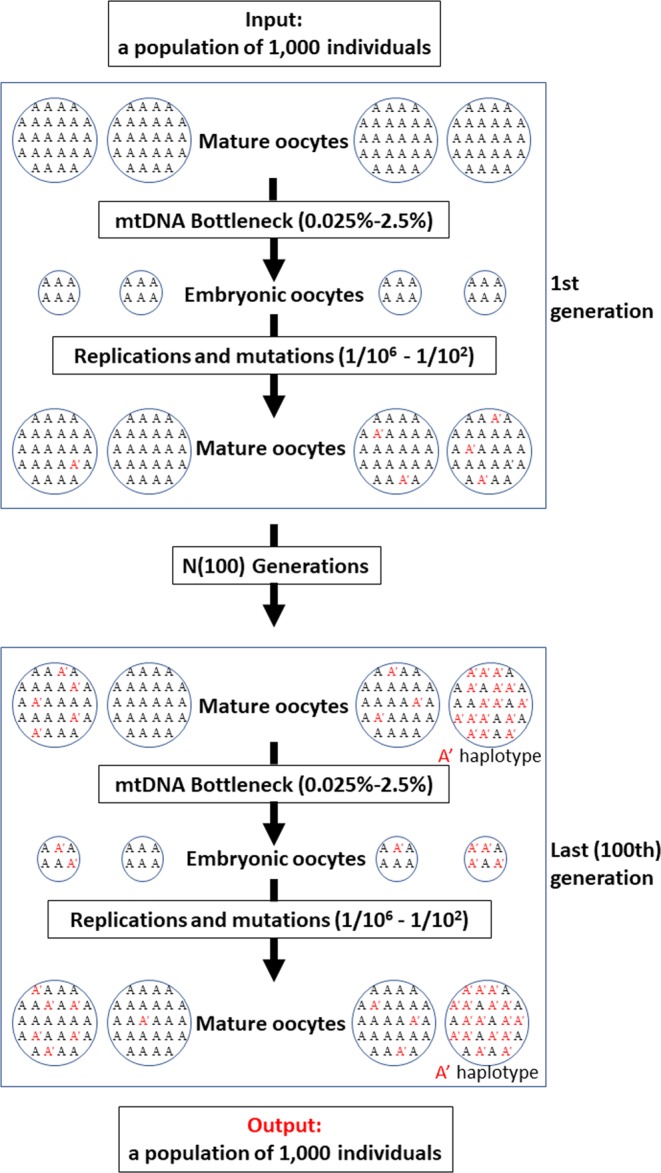
Schematic illustration of the mtDNA transmission model, also termed Multiple Molecule model (MM). Larger circles represent mature oocytes before fertilization and establishing the next generation of the population. The number of oocytes determine the size of the population (in our simulation it remained 1000, except for extinction events - as explained in the text). The letters in the circles demonstrate the composition of haplotypes (A and A′) and the ratio between them (the initial population had 100% A haplotype in all individuals). Smaller circles (and their haplotypic composition) represent the embryonic oocytes in the stage of minimum number of mtDNA molecules – the result of the mtDNA cellular bottleneck (indicated with an arrow). In our simulations, the mature oocyte mtDNA copy number was 200,000 and the bottleneck effect varied from 0.25% to 2.5%. The transition from the embryonic oocytes to mature oocytes requires replications, adding haplotype-changing mutations to the mtDNA growing population (indicated with an arrow). In our simulations, the mutation rate varied from 10^−6^ to 10^−2^, with an equal rate of back mutation. The process is repeated for N generations (N = 100) and each scenario was run 100 times. The haplotype of each mature oocyte is determined by the major haplotype (haplotype A′ is indicated). The output data include the haplotype of each mature oocyte (representing individuals in the population), the frequency of the major and the minor haplotypes, and the statistic values derived from the multiple runs. The Single Molecule model (SM), where one molecule is replicated and transmitted from one generation to the next, is straightforward and not illustrated here.

**Figure 5:**
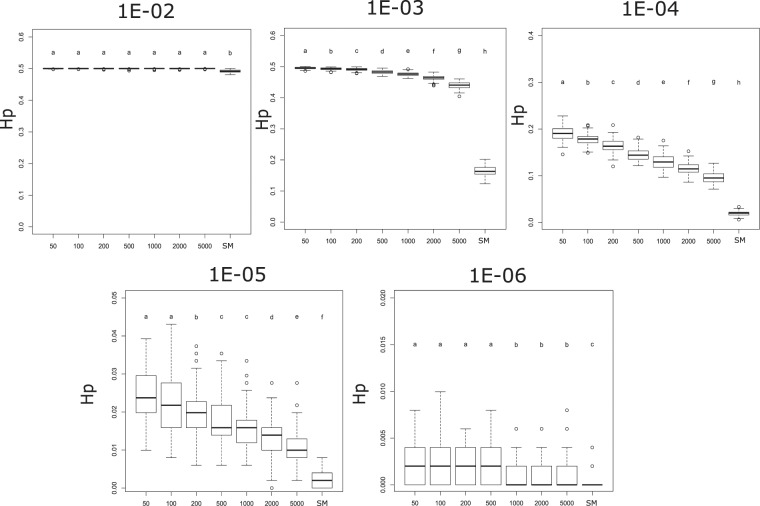
Population haplotype diversity (Hp) obtained by the Multiple Molecule model (MM) after 100 simulated generations of the 100 populations under each scenario. Each graph shows the results for a given mutation rate (from µ = 10^−2^ to µ = 10^−6^). The horizontal axis shows the different mtDNA copy number bottlenecks (from 50 to 5000 molecules) and also the Single Molecule (SM) model. Similar letters above the bar plots represent statistically non-significantly different mean values as per the post-hoc Wilcoxon signed rank test. Note the different scales of the vertical axis of the different mutation rates.

**Figure 6:**
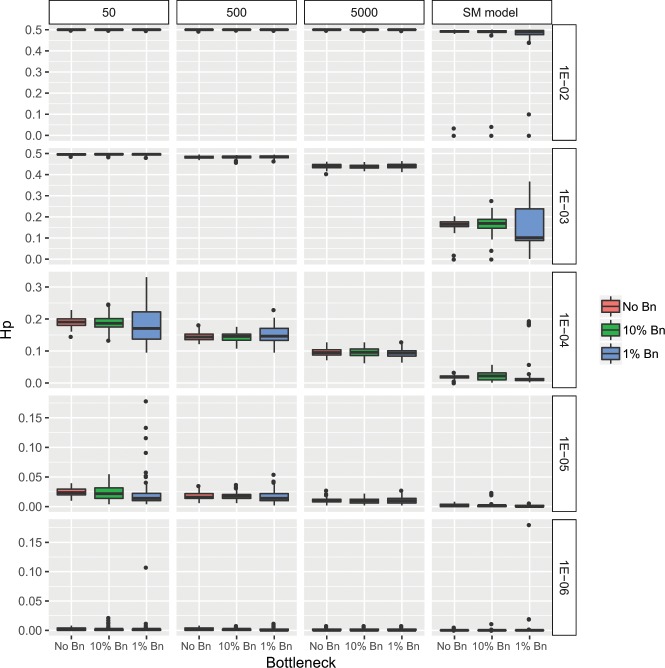
Population haplotype diversity (Hp) obtained by the Multiple Molecule model (MM) after 100 generations under different scenarios of sudden decrease in population size. Panels are ordered by the size of the mtDNA copy number bottleneck as columns (from 50 to 5000 molecules), including an additional column for the Single Molecule model (SMM), and by mutation rate as rows (from µ = 10^−2^ to µ = 10^−6^). Each panel contain three different population scenarios including no change in the population size (No Bn), a reduction of 10% of the population after 50 generations (10% Bn) and a reduction of 1% of the population after 50 generations (1% Bn). Under MM models, the bottlenecks resulted in no significant changes of haplotype diversity after FDR correction (Wilcoxon signed rank test p > 0.05) with the exception of 1% bottlenecks with a cellular bottleneck of 50 and mutation rates of 10^−5^ and 10^−6^ and with a cellular bottleneck of 500 and a mutation rate of 10^−6^. Under the SM model, all bottlenecks resulted in a significant decrease of diversity (Wilcoxon signed rank test p < 0.05) with the exception of the mutation rates of 10^−2^ and 10^−3^. Note the different scales of the vertical axis of the different mutation rates.

**Figure 7:**
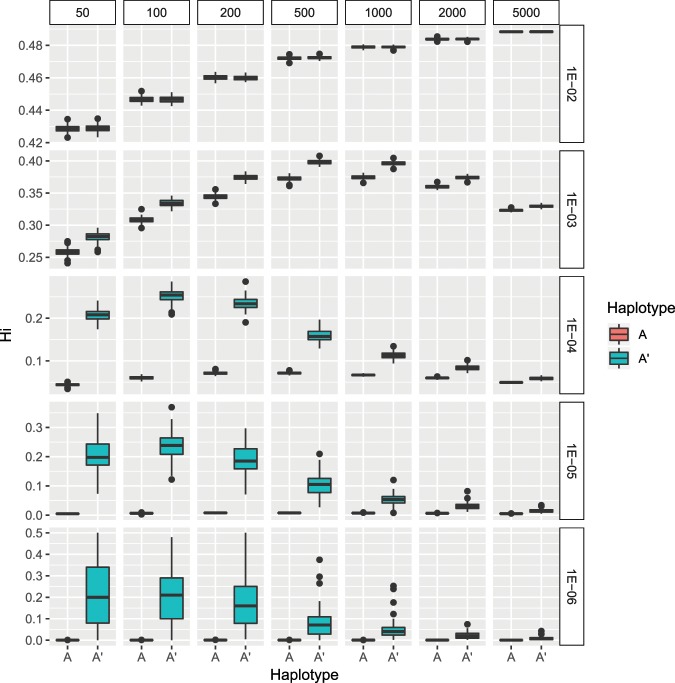
Level of individual genetic diversity per haplotype obtained by the model after 100 generations under different scenarios. Panels are ordered by the size of the mtDNA copy number bottleneck as columns (from 50 to 5000 molecules) and by mutation rate as rows (from µ = 10^−2^ to µ = 10^−6^). Each panel shows the level of individual haplotype diversity (Hi) separating the individuals by the major haplotype being the ancestral (A) or the new (A′). All pairwise comparisons of individual levels of individual haplotype diversity between the two haplotypes on each condition were significantly different (Wilcoxon signed rank test p < 0.05) with the exception of some values at 10^−2^ mutation rate.

The model of mtDNA inheritance (Fig. 4) showed that the mutation rate was the most important factor in determining the level of genetic variability (Hp) found at the end of the simulation, and thus the significantly higher levels of variability, close to the maximum possible value of 0.5, were found on the highest mutation rates (Fig. 5). However, the degree of mtDNA cellular bottleneck was also found to be a significant factor. As a result, the levels of genetic variability for each mutation rate were significantly higher at lower rates of mtDNA cellular bottleneck. As an example, mean diversity values ranged from 0.19 to 0.09 for a mutation rate of 10^−4^ depending on the strength of the cellular bottleneck. Furthermore, all the Multiple Molecule models (MMs) yielded significantly higher diversity than the Single Molecule model (SM) (Fig. 5). For the same example of the 10^−4^ mutation rate, the SM model yielded a significantly lower mean diversity of 0.02. Furthermore, the MM model buffered against loss of diversity caused by sudden decrease in population size, as no significant differences in genetic diversity were found between the non-bottleneck models and the 10% and 1% bottleneck simulations under heteroplasmic conditions (Fig. 6), although a few exceptions at extreme conditions of low mutation rates and strong mtDNA copy number bottlenecks were found. The SM model showed the opposite, as the genetic diversity after a sudden population decrease was significantly lower than without the presence of bottleneck, except for the two highest values of mutation rates (Fig. 6). Finally, the the MM models showed that the individuals with the ancestral haplotype (A) had significantly lower levels of heteroplasmy than the individuals with the alternative haplotype, except for the highest mutation rate tested (Fig. 7).

## Discussion

Using high throughput sequencing of our field study, we have provided here evidence for the evolutionary mechanisms of mtDNA heteroplasmy supported by a simulated model. Mitochondrial DNA heteroplasmy is an inevitable phenomenon since mutation in the population of mtDNA molecules will lead to heteroplasmic cells, and only a portion of the mtDNA variants would be transferred through a maternal lineage. Although mtDNA has been serving as a common tool for phylogenetic and population genetics analysis, the evolutionary mechanisms of mtDNA molecules including heteroplasmy have rarely been studied or evaluated. Sequencing techniques like Sanger di-deoxy reveal only the major haplotype, and even if heteroplasmy can sometimes be visualised, the detection and quantification of all the alternative haplotypes within individuals is not possible. Consequently, most phylogenetic and phylogeographic studies have ignored the minor haplotypes indicated by additional peaks, or at most reported it but did not use them for subsequent analysis^28,30^. Using HTS, we were able to investigate the whole population of mtDNA molecules composing an individual’s haplotypic profile by analyzing the repeat region of the mtDNA D-loop of green sea turtles. This region is not under strong selective pressure (but see^31^ for coding regions) and thus, it would allow us to improve our understanding of the mode of inheritance and evolutionary mechanisms of this molecule. We could follow the occurrence of mutations in the D-loop, the rarer SNPs^32^ as well as the more frequently occurring changes in the mtSTR short tandem repeats region^28,30^.

Our results show that heteroplasmy is very common in the green sea turtle mtSTR as it has been found in all the individuals analyzed in this study. While heteroplasmy was negligible in the region flanking the mtSTR, the frequency of minor haplotypes ranged from 10–40% of the reads within a sample for the mtSTR. This result is not unprecedented, as length heteroplasmy (e.g. variation in the number of repetitions) is more common than SNP heteroplasmy^33^. Several reasons have been proposed to explain these differences including higher selective constraints on SNP or a higher mutation rate^34^. In our case, neither the D-loop SNPs nor the mtSTR region are codifying regions and thus differences should rely on mutation rates. The mutation rate in the mtSTR region has been esteemed to be much higher (10^−2^ to 10^−4^ per generation^34–36^) than in other mitochondrial regions (10^−6^ to 10^−8^ per generation^34^). These estimates did not consider the impact of the cellular bottleneck on generating new diversity^14^ and therefore, the reported rates can overestimate the actual mutation rate. However, as all the parts of the mtDNA genome should be under the same cellular bottleneck, we can assume that the differences we observe among different mtDNA regions are due to differences in the mutation rates, even if we cannot assess either the mutation rate of the different studied regions nor the degree of the cellular bottleneck of our studied species. Given our model results, for a given cellular bottleneck, the level of heteroplasmy can vary according to the mutation rate. Consequently, it was not surprising to detect 54 different haplotypes (with a mean of 15.4 haplotypes per individual) in our study while all individuals, including the Brazilian sample, had the same sequence in the mtSTR flanking region. Heteroplasmy as the pathway to get new haplotypes also reflects mutation rate, and is therefore expected to be higher in the mtSTR region.

Our results demonstrate that, by analysing only a small number of families, we have recovered 11 out of 16 haplotypes previously reported for the whole population of Israel^30^. Furthermore, considering a previous comprehensive study in nesting females in the Mediterranean and that all our samples are from Israel, we have reported 25 new haplotypes for the Israeli population. These new haplotypes were undetected using Sanger sequencing because they were not major haplotypes for any of the analyzed individuals, but are present in the population as minor haplotypes within individuals. This includes 2 haplotypes previously thought as “orphans” (i.e., haplotypes found only in foraging areas but not in any putative nesting population of origin). The presence of orphan haplotypes is one of the shortcomings of accurate mixed stock analysis^30^. Using HTS we were able to show that these haplotypes can be present in low frequencies in heteroplasmic individuals and thus enhancing the power of mixed stock analysis.

We have also sequenced a single green sea turtle from Brazil, representing a different population. This individual had the 7_12_4_4 haplotype which is the most frequent in the Brazilian population^31^ and 7 out of the 8 most frequent minor haplotypes present in mtDNA of this turtle were major haplotypes in other Brazilian turtles^31^. These findings emphasize another aspect of heteroplasmy, suggesting that an individual is actually representative of its population, and heteroplasmic composition of each individual is not only a unique, kinship-based fingerprinting, but also carries a population signature. For instance, the recorded Israeli population major haplotypes comprise on average a striking 92.4% of the haplotypes in each individual. Consequently, heteroplasmy measured through HTS can be useful for understanding traveling and immigration patterns of sea turtles by improving mixed-stock analysis, and potentially complement methodologies like satellite tracking devices.

We found low variation among samples taken from the same individual in a time frame of 14 years. This variation was also significantly lower than the variation found among different individuals within a nest (siblings) who have inherited their mtDNA molecules from the same mother. Unlike previous studies that reported an effect of aging in increasing heteroplasmy^9^, our results indicate that a specific heteroplasmy composition can serve as fingerprinting for individuals despite aging and indicative of family clustering. For example, the siblings composing families F3, N1, and N2 share the same major haplotype. Given the difference in laying time (4 weeks), location (both Hof Gador) and sand tracks, we know that the two nests were laid by the same mother. The individual from family F3 hatched 3 years earlier, at the same location and considering our results probably has the same mother as nests N1 and N2. The event of a haplotype change is the consequence of the mode of inheritance of mtDNA, in which a limited sample of molecules from the mother compose the haplotypic variation of the offspring, enabling random effects with a great impact in a similar way to genetic drift in populations.

While it was clear from our results that the mutation rate is the main cause for generating new haplotypes, the process of generating the haplotypic composition of the female gametes, that we termed as the mtDNA cellular bottleneck, must not be overlooked. Our simulations revealed how this cellular bottleneck could work at the evolutionary level, generating new haplotypes in a population and increasing genetic diversity. Each generation is the result of two simultaneous processes. On one hand, each generation is the result of the random reproduction in which some females may reproduce more, others may reproduce less or not at all. On the other hand, once a female reproduces, the haplotypic composition of the mtDNA molecules that are transmitted to the offsprings is the result of a random sample of its own mtDNA molecules due to the cellular bottleneck. Therefore, the haplotypic composition of individuals from the same mother can be different due to the cellular bottleneck process during gametogenesis. Families N1 and N2 demonstrate how some individuals can diverge from the main cluster but, while in family N1 it is only a slight difference, family N2 demonstrates how a minor haplotype present in the mother can become the major haplotype in the offspring as a consequence of the cellular bottleneck. In this case, the individual N2_1308 major haplotype was 8_8_7_4, and the second most frequent haplotype was 8_7_7_4, which is the most frequent haplotype of the remaining siblings of the same nest. Although other important processes such as paternal leakage and *de novo mutations* are responsible for generating and maintaning mtDNA diverstiy within the cell (heteroplasmy), we found no evidence that either phenomenon contributed to increase mtDNA diversity from mother to offspring. Future studies should investigate if paternal leakage is present in sea turtles.

Unsurprisingly, the generation of new diversity within the population was related to the mutation rate, as higher mutation rates generate more new variants, but our simulations showed that the mtDNA cellular bottleneck intensity also has an important role in maintaining diversity at the population level. For similar mutation rates, stronger bottlenecks increased the impact of genetic drift at the individual level and thus the frequency of the new haplotypes within individuals can change more drastically than on milder bottlenecks. As a result, the population diversity at the end of the simulation was higher at stronger cellular bottlenecks except when very high mutation rates obscure the effect of the cellular bottleneck (Fig. 5). Consequently, a multiple molecule cellular bottleneck, accelerates the generation of new genetic diversity at the population level and thus its evolutionary advantage. This is especially evident when we compare any of the MM models to a SM model, in which all the individuals always have a single haplotype and a single molecule is replicated and transmitted to the offspring. In all cases, the genetic diversity at the end of the simulation is higher in any MM model with heteroplasmy, emphasizing its evolutionary importance.

Using the mtDNA of green turtles, we could monitor both polymorphism of the D-loop and the mtSTR on the same region. Population genetic diversity calculated using mtSTRs on a natural population has been found to be much higher than the one calculated using point mutation sequences of the same molecule^22,30^ and were shown again in our study. Considering our model, these results are not surprising as the cellular bottleneck for both markers is necessarily the same, since they are located on the same mtDNA molecule. Similarly, in agreement with our model results, it is not surprising to find fewer cases of heteroplasmy in D-loop point mutations than in mtSTRs, since regions with high mutation rates have more individual diversity for a similar cellular bottleneck (Fig. 7). Unfortunately, with our data it was not possible to get an assumption of the real mutation rates and the cellular mtDNA bottleneck effect, but it would be something very interesting to explore in future studies. While our model simulates only two haplotypes and one type of a reversible mutation for simplification, empirical data show multiple mutations and haplotypes in mtSTRs. Our results have strong implications for phylogenetics and phylogeographic studies. Since we have shown that heteroplasmy accelerates the rate of change in genetic diversity, mutation rates calculations in past studies may have been overestimated for mtDNA, thus impacting the esteem of divergence times for species or populations. Furthermore, genetic diversity can also be underestimated. For instance, the presence of minor haplotypes in the Israel population were only revealed when using high throughput sequencing.

Our study also revealed some interesting evolutionary advantages of heteroplasmy beyond increasing the genetic variability of populations. One clear observation is the significant differences between diversity rates of the individuals in which the major haplotype is the ancestral (A) or the new (A′) haplotypes. Except for some of the scenarios with the highest mutation rate (µ = 10^−2^), the new haplotype had a significantly higher individual heteroplasmy than the ancestral haplotype. The HTS results of the Mediterranean green sea turtles allowed us to observe this phenomenon in nature. Among our samples, we found individuals with five different major haplotypes, one of them (8_8_7_4) was found in only one individual. One of these haplotypes, 6_8_8_4 was found to be the most common (21.5%) in the Mediterranean and present in all Mediterranean populations^30^, thus suggesting this is the most ancient variant among the haplotypes found in our samples. The individuals carrying this haplotype had the lowest individual heteroplasmy and diversity levels (Fig. 3A), and likewise the lowest number of haplotypes (Fig. 3B), as expected, considering the results of our model (Fig. 7). The other three haplotypes also showed a correlation between the rate of individual diversity and their abundance in the Mediterranean, based on Israeli shore stranded turtles^30^. Both 6_8_9_4 and 6_9_6_4 haplotypes have the higher levels of heteroplasmy and diversity, and are much less abundant in the region (3.1% and 2.2% respectively). The fourth haplotype, 8_7_7_4, is found in 8.3% of the Mediterranean sampled individuals and has an intermediate individual heteroplasmy and diversity levels (Fig. 3). The higher heteroplasmy found in newer haplotypes indicates a possible evolutionary advantage. Mutations are a powerful evolutionary engine that creates new haplotypes, although most new haplotypes are likely to have a negative effect on the individuals bearing them if they are in a region under selection. Unlike ancestral haplotypes, new haplotypes have yet to undergo a long-term selective pressure and it is therefore safer and advantageous for an individual to keep the ancestral functional haplotypes. The fact that the number of the repeats of our study region has no evident selective effect allows for the accumulation of many minor neutral haplotypes within each individual.

We also tested the effect of heteroplasmy under the pressure of a decrease in the population size, frequently occurring in populations in general and specifically in sea turtles. Marine turtles are highly migratory vertebrates but generally exhibit some degree of philopatry to the nesting beaches. Furthermore, population sizes are generally low, especially in the Mediterranean^37^ and also genetically isolated^30^. Additionally, long distance colonization events have been described for marine turtles^23^ and thus new populations are thought to originate from a few individuals^24^. Therefore, these populations are expected to be exposed to different levels of bottlenecks due to founder effects or populations reductions, a phenomenon aggravated by anthropogenic factors such as the extreme population decline suffered by Israeli sea turtle populations during the Second World War^38^. In order to explore the impact of population bottlenecks on mtDNA evolution patterns we have used our model to study events of sudden decrease in the population size. In most cases the population decrease event did not affect the average final outcome after 100 generations (~2000 years), with a low variability among replicates. Only at lower mutation rates and smaller cellular bottlenecks, the recovery after a 99% population decrease was compromised, allowing different outcome scenarios. Nevertheless, the heteroplasmy model gave more reproducible results than the non-heteroplasmy model, showing that a population that suffers a sudden decline in size can maintain its genetic diversity stable while recuperating. Thus, heteroplasmy is not only responsible for maintaining more diversity, but also adds stability to a population’s genetic structure.

In summary, the combination of data of heteroplasmy on green turtles and the modelling of the changes in haplotype composition under different scenarios yielded new insights into the role of heteroplasmy to generate and maintain genetic mtDNA diversity within individuals and within populations. The cellular bottleneck inherent to heteroplasmy accelerates the increase of genetic diversity driven by mutation and also buffers against population bottlenecks. The combination of these two factors across an evolutionary scale likely maintain the adaptive potential of the mtDNA genome despite being uniparentally inherited. Future assessments of heteroplasmy levels in other non-model organisms and regions of the mtDNA with varying selective pressures, will extend our understanting of the importance of the results here presented for biodiversity.

## Methods

### Samples

We obtained samples of the green turtle (*Chelonia mydas*) from a captive breeding stock and natural populations. The breeding stock of the Nature and Park Authority in Michmoret, Israel has 25 turtles consisting of two families of twelve siblings each, originally hatched in the natural nesting populations Nizzanim and Hof Gador in 2002 (Israel), and a non-related individual, hatched in Hof Gador in 2004. DNA from the breeding stock (50 samples from the 25 individuals) was obtained from blood samples in 2002, 2010 and 2016. Furthermore, DNA was obtained from dead hatchlings sampled in the natural nesting population of Israel between 2001 and 2015 (n = 31). Sampling and experiments were permitted by Israeli Nature and Park Authority (NPA) and were taken as part of routine health checks. One additional sample was obtained from the south Atlantic feeding ground of Rocas Atoll. This atoll is located 600 km off the northeastern Brazilian coast and was sampled by members of Projeto Tamar in 2000 (Table 1). Samples were collected/transported under Brazilian SISBIO permit 37499-2. Tissue samples were exported/imported under CITES permits 13BR011173 and E-013161/13. All methods were carried out in accordance with each country’s relevant guidelines and regulations.

### Library preparation and sequencing

DNA extractions were performed using the DNeasy Tissue and Blood kit (Qiagen). Samples were amplified in triplicates using a modified primer pair based on the sequences from Tikochinski *et al*.^28^ (Primer F-5′ CCGATCTARCTATTYACTTCTYGTCAAACCCC-3′; Primer R 5′ CCGATCTGATACCGGCTCCTTTTATCTGAT 3′). The amplification product contained the STR region (50–70 bp) and 127 bp of its 5′ flanking region, both part of the mitochondrial DNA control region (D-loop). An Illumina tail was added to both forward and reverse primers for library preparation. The PCR reaction consisted of a 40 µL mix containing 100 ng of genomic DNA, 1X Herculase II reaction buffer, 0.4 µL of dNTPs at 25 mM each, 1 µL of each primer at 10 mM, 1% BSA, 1% DMSO, 0.8 µL of Herculase II Fusion DNA polymerase. The amplification cycle consisted of 95 °C for 3 minutes followed by 30 cycles of 94 °C for 30 seconds, 55 °C for 30 seconds, 70 °C for 30 seconds and a final extension of 70 °C for 5 minutes. Amplifications were checked in a 1% agarose gel stained with Sybr gold. All positive amplifications were purified using 1.8X CleanPCR magnetic beads (CleanNA) quantified using the dsDNA assay in Qubit 2.0 (Life Technologies), and the triplicates were pooled in an equal concentration. An 8 bp unique barcode sequence was then added to P5 and P7 regions following conditions from^39^. To minimize errors during the PCR and ensure haplotype accuracy, only high-fidelity polymerases were used in both amplification steps (Herculase II Fusion for PCR and Phusion High Fidelity for library preparation). The amplicons were purified using 0.8X CleanPCR magnetic beads (CleanNA), quantified using the dsDNA assay in Qubit 2.0 (Life Technologies) and equimolarly pooled. Blank controls consisting of ultrapure water were processed along with the samples to estimate cross-contamination. The final pooled library was characterized with a qPCR using the KAPA SYBR FAST (Kapa Biosystems) kit and also checked with the Agilent 2100 Bioanalyzer High Sensitivity DNA chip. The sequencing was performed in an Illumina MiSeq using the v3 kit (2 × 300 bp).

Reads quality control was performed with FastQC^40^ and primer integrity was checked using cutadapt^41^. Demultiplexed paired reads were merged with PEAR 0.9.5^42^ and manually checked for merging correctness. All reads that failed to merge, or with a merged fragment size smaller than the expected (350 bp) were excluded from the analysis. Haplotypes were determined using a custom Python script (available at https://bitbucket.org/Gilitiko5/turtlere/src/master/). Haplotypes were defined by the numbers of AT repeats in four (or more) tandem loci separated by a non-AT spacer, producing a four (or more) digit barcode, as described in the literature^28^. Haplotypes with a frequency of less than 1% of the reads within a sample were excluded from the analysis as previous studies have shown that index hopping^43^ might influence the assignment of reads to a sample.

To validate our mtSTR haplotypes, we used a synthethic oligonucleotide with known repeats. Previous studies have used HTS in nuclear microsatellite genotyping^44,45^ and bioinformatic processing was done by sequence abundance, given that only two options were possible for each locus (i.e. homozygous or heterozygous). In the case of mtSTR, the number of haplotypes per locus/sample is possibly higher than two, and therefore stricter controls in haplotype calling are necessary. We designed and synthesized an oligonucleotide of a 105 bp containing a common Mediterranean haplotype found in Turkey, Cyprus, and Israel, with a 6_7_6_4 repeat pattern and our PCR primers’ sequences. This synthetic DNA was sequenced in two different Illumina runs and resulting haplotypes were analysed as the other samples.

### Data analysis

Each individual was treated as a population of mtDNA molecules and thus we used classical population genetic analysis to compare individuals. For the mtSTR analysis, each ‘AT’ repeat was treated as a single mutation event and indels were added when necessary in order to have sequences of equal length. Furthermore, because of the nature of the polymorphism, only frequency-based statistics were used. We calculated the individual haplotype diversity (Hi) for each sample and we tested for structuring among individuals by calculating pairwise genetic distances (F_ST_) and performing exact tests of population differentiation as implemented in Arlequin v3.5^46^. We used Hi as a heteroplasmy measure within an individual as it accounts for the number of haplotypes within an individual and its frequency. We also calculated Hp, the haplotype diversity of the population considering only the major haplotype of all the individuals of the population. All multiple comparisons were corrected by using a false discovery rate approach^47^. The results were represented with heatmaps and dendrograms with the ‘gplots’^48^ R package^49^. Pairwise F_ST_ values obtained from Arlequin were also used to perform a Principal Coordinate Analysis (PCoA) using the GeneAlEx 6.5 software^50^ in order to distribute in a two-dimension space the genetic variability found across individuals. We tested for differences in mean pairwise F_ST_ values among samples of three different levels of relationship including (a) different samples of the same individual collected at different times (Table 1), (b) samples of different individuals within the same nest, and (c) samples of different individuals from different nests. Finally, we tested for differences in within individual haplotype diversity and within individual number of haplotypes as grouped by the major haplotype. We performed a Kruskal-Wallis test to check for global differentiation followed by a post-hoc Wilcoxon signed rank test for pairwise comparisons using R.

### Modeling mtDNA inheritance mechanisms

In order to simulate the accumulation of mutations and establishment of new haplotypes in a homogeneous population of turtles, as well as heteroplasmic rates’ measurement, a Python-based model was developed (available at https://bitbucket.org/Gilitiko5/turtlere/src/master/). The model simulates the inheritance of mtDNA across generations (as illustrated in Fig. 4), bringing into account the following variables:

- Mutation rates – the frequency of mutations that change haplotype *A* into *A*′ (and vice versa) during DNA replication. In this work, all initial populations were composed of homoplasmic *A* individuals, and the mutation rates varied from 10^−2^ to 10^−6^ per generation^34–36^. The mutation rate for the mtSTR has been previously estimated between 10^−2^ to 10^−4^, and we also added a rate within the upper range of point mutations (10^−6^).
- Number of generations – Populations were sampled after 100 generations.
- Population size – To reflect the number of individuals within the simulated population (with or without size changes through generations), we have used a constant population size of 1,000, and scenarios of sudden population size decrease to 100 or 10 after 50 generations followed by an increase back to the original size after one generation.
- Total number of mtDNA molecules:
  - In a mature oocyte. Since the exact number is not known for turtles, we have use in this work a number of mtDNA molecule estimated in other vertebrates as 200,000^51^.
  - In an embryonic oocyte. The number of mtDNA ranged from 50 to 5,000 molecules^51^

The ratio between the mature and the embryonic characterizes the mtDNA copy number bottleneck. As stated, in this work 0.025% to 2.5% mtDNA bottlenecks where simulated.

Each scenario was run 100 times in order to get an accurate average result and check its reproducibility in terms of final frequencies of the two haplotypes and heteroplasmy rate. A haplotype is determined by the majority of molecules of one haplotype (A or A′) over the other, while the heteroplasmy rate is the frequency of the minor haplotype. We used the output of the model to calculate the following parameters following the procedure described above for the sampled individuals. The haplotype diversity of the population (Hp) was calculated considering only the major haplotype of all the individuals of the population. The individual haplotype diversity (Hi) was calculated considering the haplotype composition within each individual of the population.

## Supplementary information

Supplementary File.

Supplementary Dataset 1.

## Data Availability

The raw sequence data generated for this work will be available in Dryad.
