# Author Correction: Population-specific signatures of intra-individual mitochondrial DNA heteroplasmy and their potential evolutionary advantages

**DOI:** 10.1038/s41598-020-69872-5

**Published:** 2020-07-31

**Authors:** Yaron Tikochinski, Carlos Carreras, Gili Tikochinski, Sibelle T. Vilaça

**Affiliations:** 1 Faculty of Marine Sciences, Ruppin Academic Center, Michmoret, Israel; 2 Department of Genetics, Microbiology and Statistics and IRBio, University of Barcelona, Diagonal 643, 08028 Barcelona, Spain; 3 65a Hanasi, Herzliya, Israel; 4 Leibniz Institute for Zoo and Wildlife Research, Department of Evolutionary Genetics, Berlin, Germany; 5 Berlin Center for Genomics in Biodiversity Research (BeGenDiv), Berlin, Germany; 6 Present Address: Department of Life Sciences and Biotechnology, University of Ferrara, Ferrara, Italy

Correction to: *Scientific Reports*10.1038/s41598-019-56918-6, published online 14 January 2020

The original version of this Article omitted an affiliation for Sibelle T. Vilaça. The correct affiliations for Sibelle T. Vilaça are listed below:

Present address: Department of Life Sciences and Biotechnology, University of Ferrara, Ferrara, Italy.

Leibniz Institute for Zoo and Wildlife Research, Department of Evolutionary Genetics, Berlin, Germany.

Berlin Center for Genomics in Biodiversity Research (BeGenDiv), Berlin, Germany.

Additionally, the Acknowledgements section was incomplete, where it appeared as follows:

“We thank the Israeli Nature and Park Authority and the Sea Turtle Rescue Center, headed by Dr. Yaniv Levy for acquiring samples. CC is supported by the ‘Ministerio de Ciencia Innovacion y Universidades’the ‘Agencia Estatal de Investigación/Fondo Europeo de Desarrollo Regional, Unión Europea’ (MCIU, AEI/FEDER, UE, project: ‘PopCOmics’ CTM2017-88080) and the ‘Generalitat de Cataluña’ (project: Consolidated Research Group ‘Benthic Biology and Ecology’ SGR2017-1120).”

This now reads:

“We thank the Israeli Nature and Park Authority and the Sea Turtle Rescue Center, headed by Dr. Yaniv Levy for acquiring samples. Sequencing was performed at the Berlin Center for Genomics in Biodiversity Research (BeGenDiv). We thank the sequencing lab support of Susan Mbedi and Dr. Camila Mazzoni. CC is supported by the ‘Ministerio de Ciencia Innovacion y Universidades’the ‘Agencia Estatal de Investigación/Fondo Europeo de Desarrollo Regional, Unión Europea’ (MCIU, AEI/FEDER, UE, project: ‘PopCOmics’ CTM2017-88080) and the ‘Generalitat de Cataluña’ (project: Consolidated Research Group ‘Benthic Biology and Ecology’ SGR2017-1120).”

These errors have now been corrected in the HTML and PDF versions of this Article.

