# Supplementary material for: Population-specific signatures of intra-individual mitochondrial DNA heteroplasmy and their potential evolutionary advantages
Source: Sci Rep. 2020 Jan 14;10:211. doi: 10.1038/s41598-019-56918-6 (PMC6959243; doi:10.1038/s41598-019-56918-6)
Supplement: Supplementary file 1 — Supplementary File. [file 41598_2019_56918_MOESM1_ESM.pdf]

Population-specific signatures of intra-individual mitochondrial DNA heteroplasmy and their potential evolutionary advantages

Yaron Tikochinski, Carlos Carreras, Gili Tikochinski, Sibelle T. Vilaça

Figure S1: Principal coordinate analysis (PCoA) of the Brazilian sample in relation to published mtSTR frequencies in the Mediterranean, Caribbean and Brazil. Both axes explain an accumulated 79.04% of the genetic variability found.

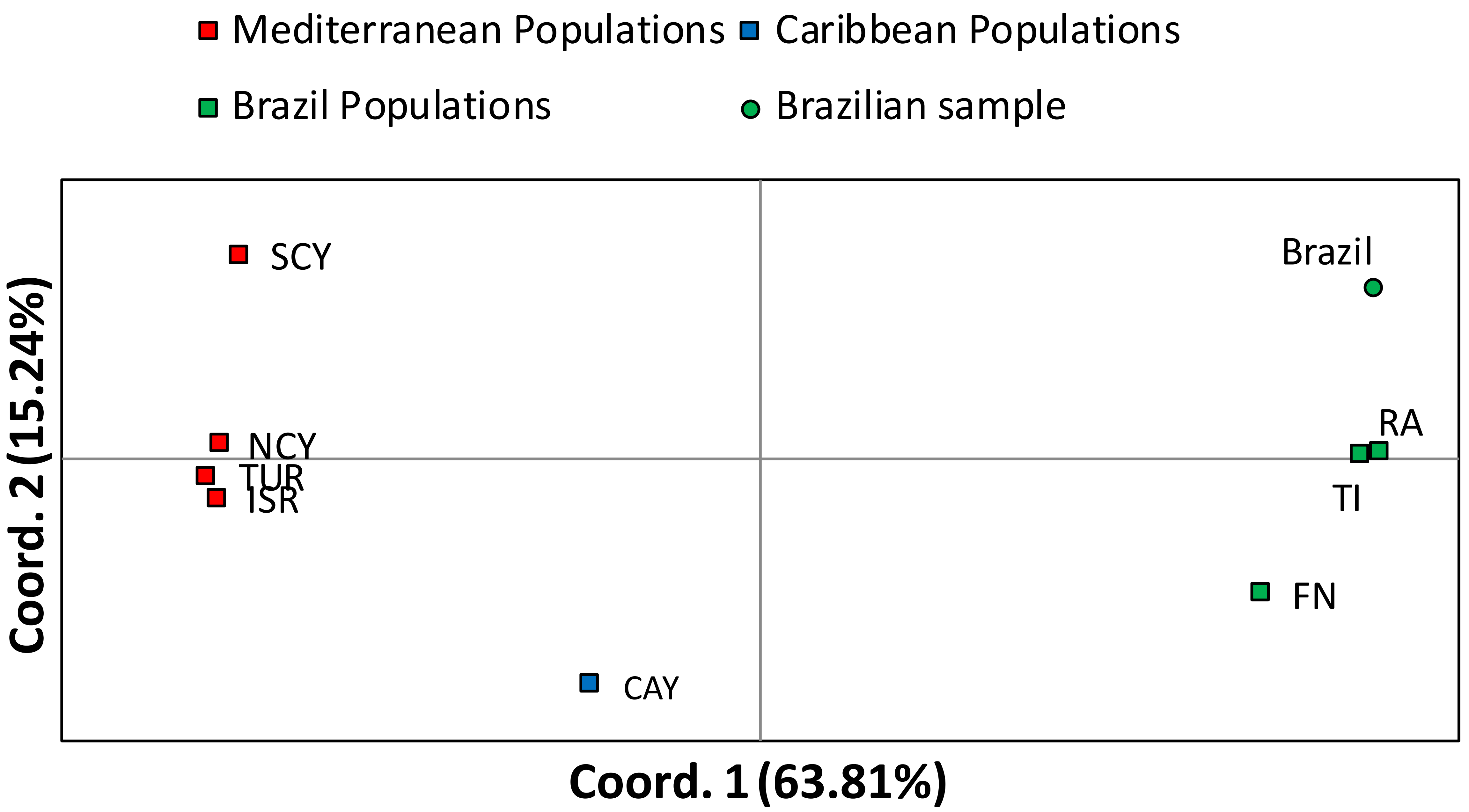

Figure S2. Diversity values of the 81 samples of known family groups including the captive animals (F1 to F3) and wild nests (N1 to N4) as detailed in Table 1. Diversity measures of the samples include A) individual haplotype diversity (Hi), and B) number of haplotypes (h). Similar letters above the bar plots represent statistically non-significantly different mean values as per the post-hoc Wilcoxon signed rank test.

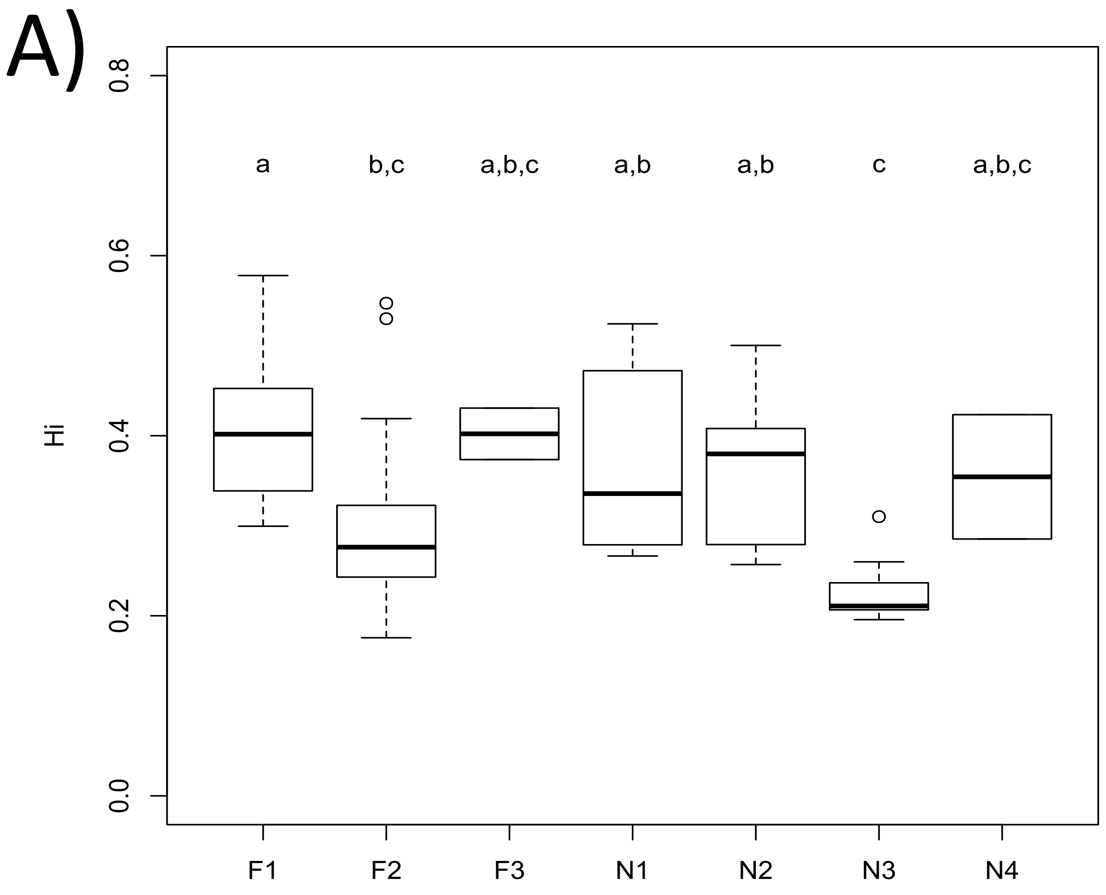

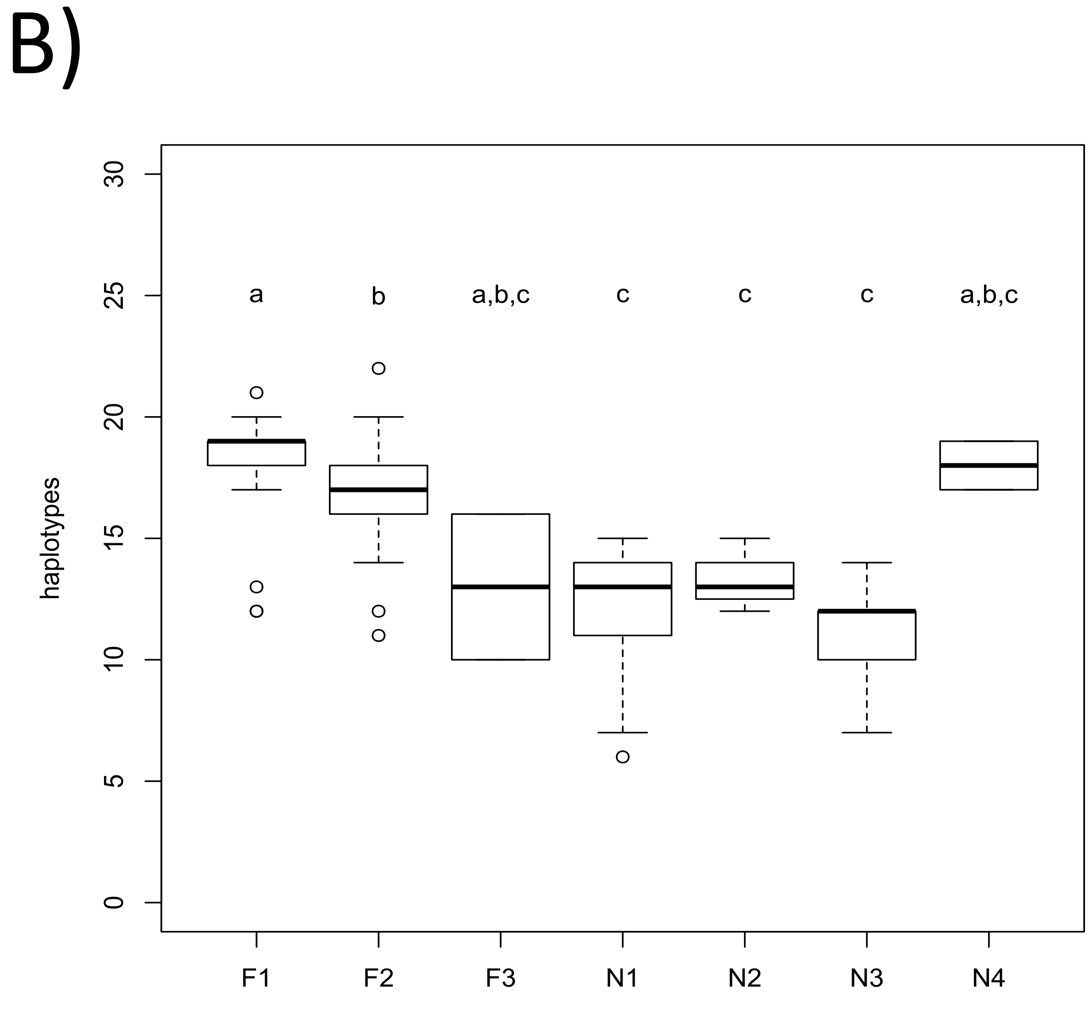

Figure S3. Pairwise differences in individual haplotype diversity (Hi). For each possible pair of samples we calculated the absolute value of the differences between their Hi values considering three different levels of relationship including i) different samples of the same individual, ii) different samples from the same nest, and iii) different samples from different nests. Similar letters above the bar plots represent statistically non-significantly different mean values as per the post-hoc Wilcoxon signed rank test.

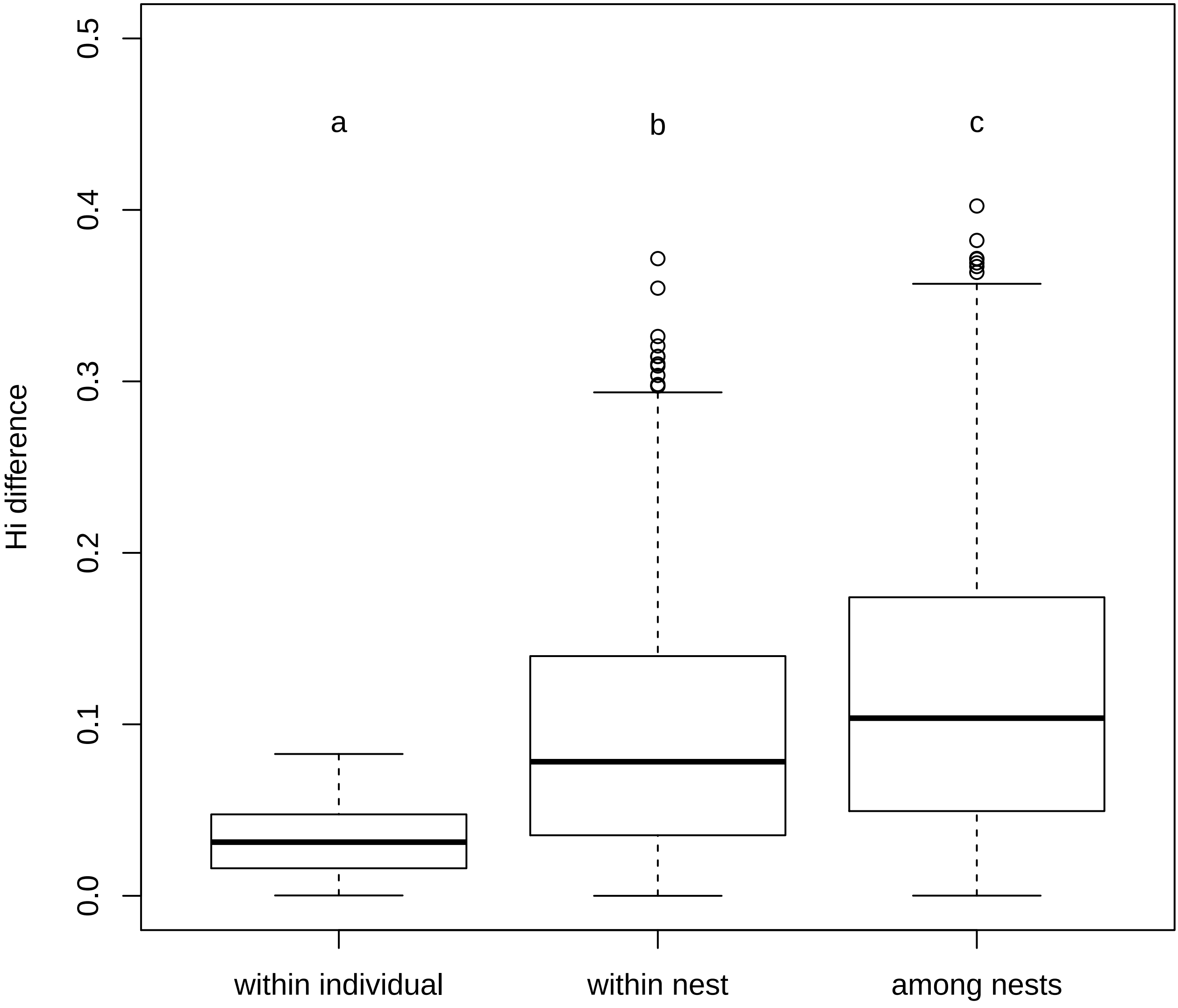

Table S1. Sampling information and general diversity indices for each sample. For each sample we include: location, Family cluster name (as in Table 1), code of the Family cluster, code of the individual, sampling date, number of reads with assigned haplotype (Reads), individual haplotypic diversity (Hi), number of haplotypes (h), the number of haplotypes previously found in Israel and the percentage of reads of haplotypes previously found in the Israeli population (% Israel).

Sample	Location	Family cluster	Code Family	Code Individual	Sampling Date	Reads	Hi	h	Haplotypes Israel	% Israel
F1_A2	Ashkelon	Family 1	F1	A2	1/6/2009	30256	0.4049	18	7	86.67
F1_A3	Ashkelon	Family 1	F1	A3	14/3/2016	156390	0.3674	19	8	86.68
F1_D3a	Ashkelon	Family 1	F1	D3	14/3/2016	171974	0.3175	19	8	94.30
F1_D3b	Ashkelon	Family 1	F1	D3	14/3/2016	11679	0.3387	12	4	94.48
F1_F2	Ashkelon	Family 1	F1	F2	1/6/2009	54615	0.4997	17	6	77.34
F1_F3	Ashkelon	Family 1	F1	F3	14/3/2016	67029	0.4778	18	7	78.71
F1_G1	Ashkelon	Family 1	F1	G1	23/10/2002	77017	0.4411	20	9	86.99
F1_N1	Ashkelon	Family 1	F1	N1	23/10/2002	64996	0.3675	21	8	89.69
F1_N3	Ashkelon	Family 1	F1	N3	14/3/2016	40995	0.4017	20	8	89.87
F1_P2	Ashkelon	Family 1	F1	P2	1/6/2009	58849	0.3856	19	8	89.33
F1_P3	Ashkelon	Family 1	F1	P3	14/3/2016	72379	0.33371	19	8	90.27
F1_Q2	Ashkelon	Family 1	F1	Q2	1/6/2009	45925	0.3239	18	7	92.49
F1_T1	Ashkelon	Family 1	F1	T1	23/10/2002	43482	0.3003	18	7	92.96
F1_T2	Ashkelon	Family 1	F1	T2	1/6/2009	57389	0.3144	19	7	92.59
F1_T3	Ashkelon	Family 1	F1	T3	14/3/2016	66720	0.2995	19	8	93.64
F1_V1	Ashkelon	Family 1	F1	V1	23/10/200	11151	0.5779	13	4	71.35

					2					
F1_V2	Ashkelon	Family 1	F1	V2	1/6/2009	95938	0.5392	19	7	75.42
F1_X1	Ashkelon	Family 1	F1	X1	23/10/2002	95654	0.4405	19	8	88.16
F1_X2	Ashkelon	Family 1	F1	X2	1/6/2009	71113	0.4051	20	8	88.67
F1_X3	Ashkelon	Family 1	F1	X3	14/3/2016	86276	0.4113	19	8	88.52
F1_Z1	Ashkelon	Family 1	F1	Z1	23/10/2002	126163	0.4525	17	6	83.32
F1_Z2	Ashkelon	Family 1	F1	Z2	1/6/2009	72078	0.4859	19	7	81.24
F1_Z3	Ashkelon	Family 1	F1	Z3	14/3/2016	6119	0.5041	12	5	81.57
F1_AA1	Ashkelon	Family 1	F1	AA1	23/10/2002	97391	0.3463	18	7	91.72
F1_AA2	Ashkelon	Family 1	F1	AA2	1/6/2009	49504	0.3461	20	8	91.84
F2_B1	Giv'at Olga	Family 2	F2	B1	23/10/2002	57696	0.419	17	9	83.53
F2_B1b	Giv'at Olga	Family 2	F2	B1b	23/10/2002	12399	0.3693	11	6	85.49
F2_C1	Giv'at Olga	Family 2	F2	C1	23/10/2002	78629	0.2627	17	9	96.10
F2_C3_b	Giv'at Olga	Family 2	F2	C3_b	14/3/2016	195180	0.237	17	9	96.82
F2_E3	Giv'at Olga	Family 2	F2	E3	14/3/2016	28795	0.1756	12	6	97.74
F2_H1	Giv'at Olga	Family 2	F2	H1	23/10/2002	75336	0.2779	18	9	96.42
F2_H3	Giv'at Olga	Family 2	F2	H3	14/3/2016	47100	0.2326	14	7	97.27
F2_J2	Giv'at Olga	Family 2	F2	J2	1/6/2009	76066	0.2762	17	10	96.49
F2_J3	Giv'at Olga	Family 2	F2	J3	14/3/2016	63905	0.2536	15	8	96.77

F2_K1	Giv'at Olga	Family 2	F2	K1	23/10/2002	20777	0.3037	17	9	96.95
F2_K3	Giv'at Olga	Family 2	F2	K3	14/3/2016	183591	0.221	18	9	97.04
F2_K3b	Giv'at Olga	Family 2	F2	K3b	14/3/2016	98184	0.2265	14	7	97.28
F2_L1	Giv'at Olga	Family 2	F2	L1	23/10/2002	83874	0.3058	18	9	96.39
F2_L3	Giv'at Olga	Family 2	F2	L3	14/3/2016	116883	0.268	17	9	96.76
F2_R2	Giv'at Olga	Family 2	F2	R2	1/6/2009	77530	0.3395	18	10	92.62
F2_S1	Giv'at Olga	Family 2	F2	S1	23/10/2002	70478	0.2327	16	9	97.33
F2_S2	Giv'at Olga	Family 2	F2	S2	1/6/2009	72495	0.2981	18	11	96.26
F2_U1	Giv'at Olga	Family 2	F2	U1	23/10/2002	70202	0.249	16	8	96.83
F2_U2	Giv'at Olga	Family 2	F2	U2	1/6/2009	88633	0.2596	17	9	97.01
F2_W2	Giv'at Olga	Family 2	F2	W2	1/6/2009	158538	0.5472	20	10	92.03
F2_W3	Giv'at Olga	Family 2	F2	W3	14/3/2016	39416	0.53	17	9	93.36
F2_Y1	Giv'at Olga	Family 2	F2	Y1	23/10/2002	156784	0.3472	22	11	96.33
F2_Y2	Giv'at Olga	Family 2	F2	Y2	1/6/2009	69214	0.2782	17	10	96.62
F3_O2	Hof Gador	Family 3	F3	O2	1/6/2009	122096	0.4307	16	9	86.50
F3_O3	Hof Gador	Family 3	F3	O3	14/3/2016	97797	0.3735	10	6	89.04
N1_1109	Hof Gador	Nest 1	N1	1109	23/8/2008	117513	0.2835	15	7	97.24

N1_1110	Hof Gador	Nest 1	N1	1110	23/8/2008	76643	0.2664	13	6	97.13
N1_1112	Hof Gador	Nest 1	N1	1112	23/8/2008	63857	0.5204	15	8	73.43
N1_1113	Hof Gador	Nest 1	N1	1113	23/8/2008	58821	0.278	13	6	97.25
N1_1116	Hof Gador	Nest 1	N1	1116	23/8/2008	609	0.4722	6	5	97.70
N1_1117	Hof Gador	Nest 1	N1	1117	23/8/2008	1874	0.3357	7	5	97.39
N1_1118	Hof Gador	Nest 1	N1	1118	23/8/2008	50987	0.2788	14	6	97.07
N1_1119	Hof Gador	Nest 1	N1	1119	23/8/2008	12364	0.5243	11	5	94.82
N1_11120	Hof Gador	Nest 1	N1	11120	23/8/2008	47429	0.368	13	5	96.29
N2_1302	Hof Gador	Nest 2	N2	1302	17/9/2008	21168	0.3825	12	5	95.37
N2_1303	Hof Gador	Nest 2	N2	1303	17/9/2008	60846	0.3868	14	5	97.31
N2_1304	Hof Gador	Nest 2	N2	1304	17/9/2008	78062	0.2931	13	6	95.60
N2_1305	Hof Gador	Nest 2	N2	1305	17/9/2008	50820	0.2589	12	5	97.43
N2_1306	Hof Gador	Nest 2	N2	1306	17/9/2008	45282	0.3489	14	7	97.52
N2_1307	Hof Gador	Nest 2	N2	1307	17/9/2008	59434	0.2569	12	5	97.48
N2_1308	Hof Gador	Nest 2	N2	1308	17/9/2008	19368	0.4788	14	7	18.81
N2_1309	Hof Gador	Nest 2	N2	1309	17/9/2008	56339	0.3798	14	6	97.41
N2_1310	Hof Gador	Nest 2	N2	1310	17/9/2008	52392	0.2651	13	6	97.32
N2_1311	Hof Gador	Nest 2	N2	1311	17/9/2008	48261	0.5003	13	6	82.74
N2_1312	Hof Gador	Nest 2	N2	1312	17/9/2008	61441	0.4292	15	7	83.79
N3_3067	Hof Gador	Nest 3	N3	3067	30/8/2013	43180	0.231	14	9	94.72
N3_3068	Hof Gador	Nest 3	N3	3068	30/8/2013	25726	0.2062	12	8	95.20
N3_3069	Hof Gador	Nest 3	N3	3069	30/8/2013	5978	0.2109	7	5	94.55
N3_3070	Hof Gador	Nest 3	N3	3070	30/8/2013	22221	0.1957	11	7	95.75
N3_3073	Hof Gador	Nest 3	N3	3073	30/8/2013	28450	0.2088	10	6	95.44
N3_3074	Hof Gador	Nest 3	N3	3074	30/8/2013	21567	0.2366	13	8	95.09
N3_3076	Hof Gador	Nest 3	N3	3076	30/8/2013	29824	0.2066	12	8	95.27
N3_3078	Hof Gador	Nest 3	N3	3078	30/8/2013	11023	0.2599	12	8	95.56
N3_3080	Hof Gador	Nest 3	N3	3080	30/8/2013	25600	0.3101	10	6	87.54

N4_3230	Hof Gador	Nest 4	N4	3230	16/9/2015	22172	0.4234	17	9	94.95
N4_3231	Hof Gador	Nest 4	N4	3230	16/9/2015	27603	0.2853	19	10	94.80
